# Beneficial effect of repeated participation in breast cancer screening upon survival

**DOI:** 10.1177/09691413231186686

**Published:** 2023-07-12

**Authors:** Stephen W Duffy, Amy Ming-Fang Yen, Laszlo Tabar, Abbie Ting-Yu Lin, Sam Li-Sheng Chen, Chen-Yang Hsu, Peter B Dean, Robert A Smith, Tony Hsiu-Hsi Chen

**Affiliations:** 1 Centre for Prevention, Detection and Diagnosis, Wolfson Institute of Population Health, 4617 Queen Mary University of London, London, UK; 2 School of Oral Hygiene, College of Oral Medicine, 38032 Taipei Medical University, Taipei, Taiwan; 3 Falun Central Hospital, Falun, Sweden; 4 Institute of Epidemiology and Preventive Medicine, College of Public Health, 33561 National Taiwan University, Taipei, Taiwan; 5 Master of Public Health Program, College of Public Health, 33561 National Taiwan University, Taipei, Taiwan; 6 University of Turku, Turku, Finland; 7 American Cancer Society, Atlanta, GA, USA

**Keywords:** breast cancer, mammography, survival

## Abstract

**Objectives:**

The benefit of mammography screening in reducing population mortality from breast cancer is well established. In this paper, we estimate the effect of repeated participation at scheduled screens on case survival.

**Methods:**

We analysed incidence and survival data on 37,079 women from nine Swedish counties who had at least one to five invitation(s) to screening prior to diagnosis, and were diagnosed with breast cancer between 1992 and 2016. Of these, 4564 subsequently died of breast cancer. We estimated the association of survival with participation in up to the most recent five screens before diagnosis. We used proportional hazards regression to estimate the effect on survival of the number of scheduled screens in which subjects participated prior to the diagnosis of breast cancer.

**Results:**

There was successively better survival with an increasing number of screens in which the subject participated. For a woman with five previous screening invitations who participated in all five, the hazard ratio was 0.28 (95% confidence interval (CI) 0.25–0.33, *p* < 0.0001) compared to a woman attending none (86.9% vs 68.9% 20-year survival). Following a conservative adjustment for potential self-selection factors, the hazard ratio was 0.34 (95% CI 0.26–0.43, *p* < 0.0001), an approximate three-fold reduction in the hazard of dying from breast cancer.

**Conclusion:**

For those women who develop breast cancer, regular prior participation in mammography screening confers significantly better survival.

## Introduction

The randomised trials of mammography screening have shown a substantial and significant reduction in breast cancer mortality with the offer of screening. This can be seen in the publications of the individual trials and in numerous official reviews of the breast cancer screening evidence.^1–5^ Since the trials were published in the late twentieth century, mammography screening has become policy in many middle- and high-income countries. Evaluation of service screening has shown that in routine healthcare, mammography screening is achieving at least the mortality benefits expected from the trials.^6,7^ It is also clear that measuring the effect of an invitation to screening underestimates the benefit of participating in screening.^7^

A recent analysis of population screening data in Sweden found that participation in two successive scheduled screening mammograms conferred a greater reduction in the risk of breast cancer mortality than did participation in only one of the two.^8^ For an individual breast cancer patient, it seems reasonable to assume that the only screen that can prevent death from breast cancer is the screen that detects the cancer. However, it is also reasonable to consider that with more regular participation in screening, it will be more likely that one of the screening examinations will detect the cancer and that the cancer will have less time to grow between screening examinations.

In this study, therefore, we investigated the effect of the number of scheduled screens in which each woman participated prior to her breast cancer diagnosis, and the association of this participation with individual case survival from breast cancer. We used comprehensive data on screening history and subsequent survival from cancers diagnosed in nine counties in Sweden with an average of 19 years of follow-up from diagnosis.

## Data and methods

According to national recommendations, women in Sweden are invited to screening with a letter including a prebooked appointment. During the study period, between 1992 and 2016, national policy was to screen women aged 40–54 years every 18 months and those aged 55–69 years every 24 months. However, decisions on exact screening age ranges are devolved to individual counties. In addition, dates of introduction of screening varied by county. Participation rates vary but are generally high, from around 70%–90%.^9^

Data were available for 37,079 breast cancer patients diagnosed in the nine counties during the period of observation, among whom 4564 breast cancer deaths occurred subsequently. For each breast cancer diagnosed in each county's period of observation, we obtained data on previous screening history, and subsequent death (or not) from breast cancer. We had follow-up data on cancer incidence and death to 31 December 2019. We extracted data on participation (or not) in response to the five most recent screening invitations prior to breast cancer diagnosis. For women with fewer than five previous screening invitations (see below) we obtained data on participation in the total number of prior screening episodes available. Breast cancer incidence data were obtained from the regional oncology centres of the Northern, Uppsala-Örebro, and Stockholm-Gotland health care regions. Data on subsequent death from breast cancer were provided by the Swedish Cause of Death Register (Swedish National Board of Health and Welfare). These data were linked to population screening data on the invitation to and participation in screening mammography, prospectively collected by Sectra Medical Systems, Linkӧping, Sweden.

The number of prior invitations to screening necessarily depends on the age at diagnosis. For example, in a county where 2-yearly screening starts at age 50, a woman aged 54 at diagnosis would be expected to have had two or three prior invitations, whereas a woman aged 60 would be expected to have had at least five prior invitations. For breast cancer patients with 1, 2, 3, 4 and 5 prior invitations to screening, we tabulated the numbers of screens participated in by breast cancer cases and deaths, and the corresponding 20-year survival. Formal comparisons of survival with respect to numbers of screens participated in were carried out using Cox proportional hazards regression with time-varying covariates, i.e., cumulative numbers of screens,^10^ yielding hazard ratios and 95% confidence intervals (CI).

The effect of repeated participation was expressed as the relative hazard of death from breast cancer for the number of screens participated in, with participation in no screens as the baseline category, calculated from the Cox regression. In addition to regular screening making early detection more likely and giving a tumour less time to grow, there is a possibility that those who elect not to participate in screening have poorer outcomes a priori as observed in the randomised trials of screening.^1,2^ In the long-term follow-up of the Swedish Two-County trial, the fatality from cancers in non-attenders was 77/140 = 0.55, whereas in the unscreened controls it was 332/755 = 0.44, a relative risk of 1.20. We corrected for this potential self-selection using the method of Duffy et al.,^11^ which gives a corrected estimate based on the proportion of women accepting the invitation and participating in the screening, the uncorrected estimate, and the relative risk of breast cancer death for non-participants compared to an uninvited population.

This study was approved by the ethics committee of Uppsala University (registration number 2017/147).

## Results

 Table 1 shows the nine Swedish counties (Stockholm, Dalarna, Värmland, Örebro, Västmanland, Gävleborg, Västernorrland, Västerbotten, and Norrbotten) for which we had data, with periods of observation and the target age range for screening.

**Table 1.** Years of observation and screening age ranges by county.

County	Years of observation	Screening age range (years)	Number of breast cancers	Number of breast cancer deaths
Dalarna	1993–2016	40–69	3154	360
Stockholm	1992–2016	50–69	17522	2184
Värmland	1993–2016	50–69	2354	297
Örebro	1992–2016	50–69	2319	339
Västmanland	1992–2016	40–69	3002	392
Gävleborg	2001–2016	40–69	2182	193
Västernorrland	1997–2016	40–69	2513	300
Västerbotten	1997–2016	50–69	1790	220
Norrbotten	1997–2016	40–69	2243	279

Of the 37,079 breast cancer patients, Table 2 shows the numbers participating in screening by the number of invitations. Depending on the number of invitations, 58%–73% (average 65%) participated in all scheduled screens, and 73%–96% (average 91%) participated in at least one. Table 3 gives the corresponding numbers of breast cancer deaths and 20-year survival rates. For those participating in all screens, survival rates ranged from 82.7% to 86.9% (average 85.3%). For those participating in no screens, survival ranged from 59.1% to 77.6% (average 70.5%). For each number of invitations, the difference in survival by number of screens participated in was significant (*p* < 0.0001) in all cases compared to women participating in no screens.

**Table 2.** Attendance at screens by number of invitations prior to diagnosis for 37,079 cancer patients.

Number of invitations	Number of screens attended (% of total patients in each invitation group)						
	0	1	2	3	4	5	Total
1	937 (27)	2585 (73)	-	-	-	-	3522
2	576 (15)	826 (22)	2353 (63)	-	-	-	3755
3	469 (12)	381 (9)	762 (19)	2413 (60)	-	-	4025
4	354 (8)	292 (6)	384 (8)	857 (19)	2631 (58)	-	4518
5	937 (4)	681 (3)	872 (4)	1350 (6)	3346 (16)	14073 (66)	21259
∑1-5	3273 (9)	4765 (13)	4371 (12)	4620 (12)	5977 (16)	14073 (38)	37079

**Table 3.** Numbers of breast cancer deaths by number of invitations prior to diagnosis and numbers of screens attended, with 20-year survival rates.

Number of invitations	Breast cancer deaths by number of screens attended (20-year survival, %)						
	0	1	2	3	4	5	Total
1	193 (77.6)	357 (84.9)	-	-	-	-	550 (83.0)
2	160 (68.2)	135 (80.6)	357 (82.7)	-	-	-	652 (80.1)
3	119 (67.7)	65 (79.0)	121 (80.3)	322 (83.5)	-	-	627 (80.8)
4	119 (59.1)	52 (77.1)	51 (78.3)	93 (86.4)	318 (84.7)	-	633 (82.1)
5	216 (68.9)	118 (72.4)	114 (76.4)	135 (85.2)	353 (83.4)	1166 (86.9)	2102 (84.7)
∑1-5	807 (70.5)	727 (82.0)	643 (81.4)	550 (84.4)	671 (84.1)	1166 (86.9)	4564 (83.2)

**Figure 1.**
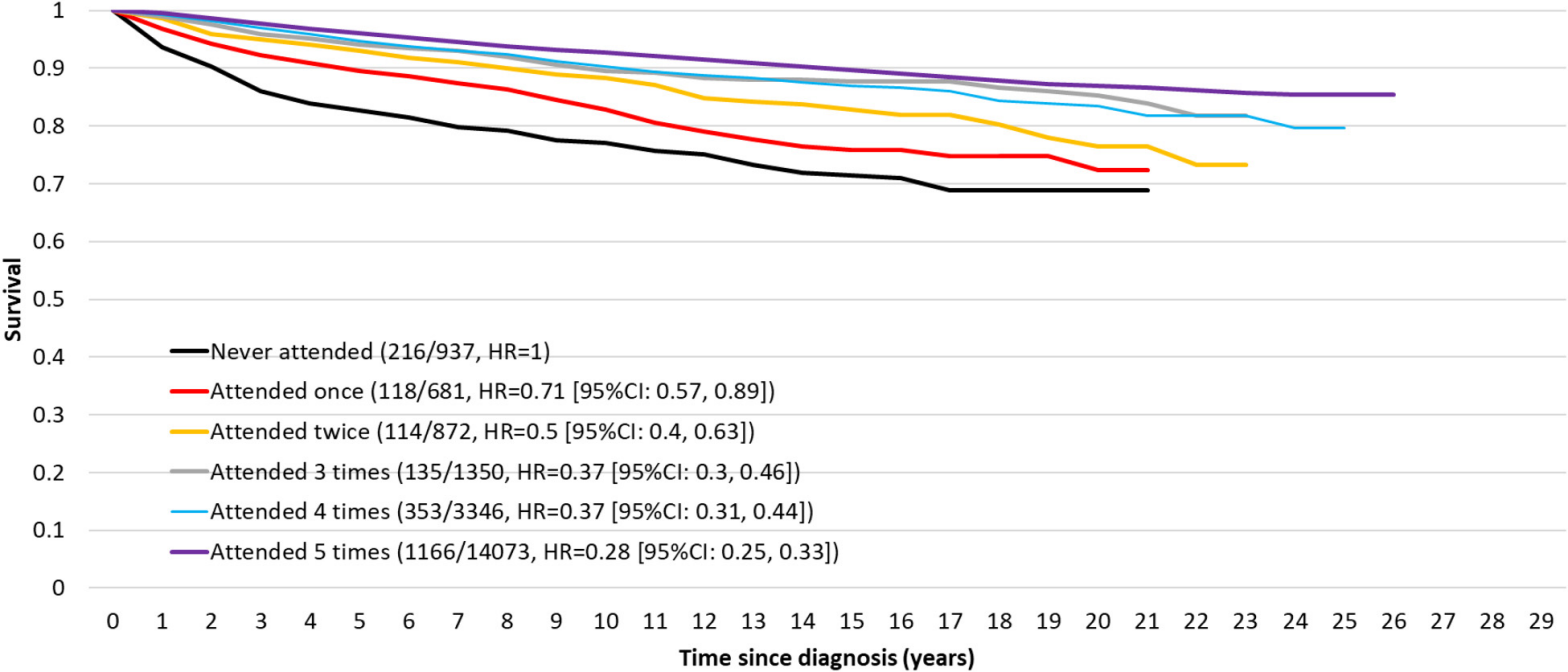
Survival by the number of screens attended for those with five previous invitations.

These results are illustrated in Figure 1 for the case of women diagnosed with breast cancer who had five previous screening invitations. There is successively better survival with an increasing number of screens participated in. For a woman participating in all five, the hazard ratio was 0.28 (95% CI 0.25–0.33, *p* < 0.0001). Table 4 shows the corresponding hazard ratios for each county separately.

**Table 4.** Estimated hazard ratios by screening participation for each county separately and for all counties combined, for women with five prior invitations to screening.

County	Hazard ratio (95% CI) for participation in				
	1 screen	2 screens	3 screens	4 screens	5 screens
Stockholm	0.75 (0.55–1.02)	0.55 (0.40–0.75)	0.49 (0.37–0.66)	0.52 (0.42–0.66)	0.36 (0.29–0.44)
Värmland	0.72 (0.28–1.88)	0.58 (0.21–1.64)	0.26 (0.10–0.70)	0.11 (0.04–0.30)	0.16 (0.08–0.32)
Örebro	0.49 (0.18–1.30)	0.30 (0.13–0.71)	0.24 (0.10–0.55)	0.20 (0.10–0.40)	0.18 (0.10–0.33)
Västmanland	0.93 (0.44–1.97)	0.52 (0.25–1.10)	0.24 (0.11–0.52)	0.26 (0.14–0.48)	0.20 (0.11–0.44)
Dalarna	0.39 (0.21–0.71)	0.36 (0.16–0.81)	0.57 (0.26–1.28)	0.44 (0.20–0.99)	0.38 (0.27–0.55)
Gävleborg	0.11 (0.01–0.85)	0.20 (0.04–0.91)	0.05 (0.01–0.43)	0.23 (0.10–0.54)	0.09 (0.04–0.20)
Västernorrland	0.41 (0.18–0.91)	0.21 (0.09–0.52)	0.17 (0.08–0.36)	0.18 (0.11–0.31)	0.13 (0.09–0.21)
Västerbotten	0.12 (0.04–0.42)	0.13 (0.03–0.56)	0.19 (0.06–0.68)	-*	0.17 (0.09–0.31)
Norrbotten	0.78 (0.34–1.80)	0.61 (0.27–1.39)	0.19 (0.07–0.48)	0.21 (0.10–0.45)	0.17 (0.09–0.34)
All counties combined	0.71 (0.57–0.89)	0.50 (0.40–0.63)	0.37 (0.30–0.46)	0.37 (0.31–0.44)	0.28 (0.25–0.33)

*insufficient data for estimation (27 cases, no breast cancer deaths).

 Table 5 shows the overall hazard ratios for all counties combined, in women who had received five invitations to screening, unadjusted and adjusted for potential self-selection factors using the method of Duffy et al.^11^ We used the relative risk for non-participants of 1.20 derived from the Swedish Two-County study (see ‘Methods’ section above), and the proportion of participants of 91% (see Table 2) to make the correction. Even after this conservative adjustment, there was a clear and highly significant reduction in hazard of breast cancer death with increasing number of screens participated in. For participation in all five screens, the hazard ratio was 0.34 (95% CI 0.26–0.43, *p* < 0.0001).

**Table 5.** Hazard ratios by number of screens in which the women participated, for women who had received five invitations, unadjusted and adjusted for potential self-selection factors.

Number of screens participated in	Hazard ratio (95% CI)	
	Unadjusted	Adjusted
0	1.00 (-)	1.00 (-)
1	0.71 (0.57–0.89)	0.87 (0.67–1.12)
2	0.50 (0.40–0.63)	0.61 (0.45–0.83)
3	0.37 (0.30–0.46)	0.45 (0.34–0.60)
4	0.37 (0.31–0.43)	0.45 (0.35–0.58)
5	0.28 (0.25–0.33)	0.34 (0.26–0.43)

## Discussion

We analysed disease-specific survival in relation to repeated participation in screening for 37,079 breast cancers diagnosed in nine counties in Sweden. We found a greater reduction in the hazard of breast cancer death with increasing number of screens in which the women participated. Of those with five prior invitations to screening, the relative hazard of breast cancer death for those participating in all five screens was 0.28 (95% CI 0.25–0.33) compared to women who had participated in none, a 72% reduction in the risk of dying from breast cancer. Even after conservative adjustment for potential self-selection factors, there was a highly significant 66% reduction in hazard, with a hazard ration of 0.34 (95% CI 0.26–0.43).

Our finding of successively better survival with a number of screens participated in agrees with the results observed by Seiffert et al. with respect to breast screening in Germany,^12^ who observed an association between smaller tumour sizes at diagnosis in patients with family history who had a higher number of prior mammograms. In a study of elderly women in the USA, Badgwell et al. found a trend of increasing survival with the regularity of mammography use.^13^ Also in the USA, Randolph et al. found a decreasing trend in the proportion of late-stage disease with the number of prior mammograms.^14^ In Australia, Kou et al.^15^ found that regular mammography was associated with a decreased risk of late stage, grade 3 or hormone receptor negative cancers. Liu et al. found that in breast cancer patients with four prior mammograms compared with one, there were fewer stage 3 and 4 cancers and correspondingly fewer mastectomies, lower rates of chemotherapy and more patients receiving breast-conserving surgery.^16^ Thus, regular participation may also reduce the need for, and risks associated with more aggressive therapy.

There is a policy implication of the successively greater hazard reduction with an increasing number of screens in which a woman participates. This implies that screening programmes with wider age ranges and/or shorter interscreening intervals, which are therefore characterised by having larger numbers of screening episodes available for participation, will in turn confer better breast cancer outcomes overall.

In terms of limitations, it should be noted that these results are for case survival, not population mortality. However, it is worth noting that the results are consistent with a greater reduction in population mortality observed for those who participated in both their most recent scheduled screens compared to those who participated in only one.^8^ Collation of population mortality data is in progress and results will be reported in a future paper. It should be noted that direct comparison of groups with different numbers of invitations is confounded by age, breast cancer risk and perhaps by general health status, since to have had five invitations a woman has to have lived for at least eight years beyond the lower age limit for screening. For this reason, our focus in this analysis was comparison within the population that had received five invitations. This comparison within the population is not confounded.

In conclusion, we found that regular participation in mammography screening was associated with a substantial and significant improvement in survival from breast cancer, with data showing successively increasing survival with a number of screens in which the women participated. Most women will not develop breast cancer in their lifetime. These results indicate that for those who do, regular participation in screening considerably improves the probability of surviving it. The distinction between the general, population benefit of mammography screening, and the individual benefit of regular participation in mammography screening should be clearly articulated in breast cancer screening messaging and decision aids.
